# Cancer and Indigenous Populations: Time to End the Disparity

**DOI:** 10.1200/JGO.19.00379

**Published:** 2020-01-13

**Authors:** Eva Segelov, Gail Garvey

**Affiliations:** ^1^ Monash University and Monash Health, Clayton, Victoria, Australia; ^2^ Menzies School of Health Research, Darwin, Northern Territory, Australia

*“Indigenous peoples are at higher risk for cancers and other diseases and have worse health outcomes than non-Indigenous groups but this is only the beginning of our understanding of the problem.”* Tedros Adhanam Ghebreyesus, World Health Organization, 2018

This first-ever Special Issue of *Journal of Global Oncology* is dedicated to research and commentary on the hidden problem of cancer in Indigenous populations. Worldwide, there are approximately 370 million Indigenous people, spread across 90 countries.^1^ Indigenous peoples and communities are culturally diverse and are distinct from the dominant societies in which they live. In considering the diversity of Indigenous peoples, the United Nations Permanent Forum on Indigenous Issues provides several characteristics that guide their identification: self-identification as Indigenous peoples at the individual level and accepted by the community as its member; historical continuity with precolonial and/or presettler societies; strong link to territories and surrounding natural resources; social, economic, or political systems, language, culture, and beliefs that are distinct from nondominant groups of society; and resolve to maintain and reproduce their ancestral environments and systems as distinctive peoples and communities.^1^

The health of Indigenous peoples is intimately linked to the social, structural, and political environments in which they live. It is a matter of shame that Indigenous people constitute 5% of the total global population yet account for approximately 15% of the world’s extreme poor, according to the World Bank.^2^ Health disparities are well documented in Indigenous populations, in whom cancer is increasingly a priority as deaths from acute infections and other imminent health dangers become controlled.

Accordingly, this Special Series comprises articles sourced around the globe, focused on three domains: (1) the epidemiology and disease and treatment patterns of cancer in different Indigenous populations; (2) the cultural impact of Indigenous culture on the conduct of cancer research and care; and—last but by no means least— 3) the empowerment and engagement of Indigenous researchers and communities in setting their own agenda for improving cancer outcomes.

## Epidemiology and Disease and Treatment Patterns

In some regions, Indigenous peoples are unrecognized and uncounted. The rights of Indigenous peoples, including the right to be counted in population and health data collection, have become a focus of the United Nations.^3,4^ Without robust data, the true burden and disparities in cancer in Indigenous populations will remain obscured. The article by Diaz et al^5^ provides the first global report on the collection and reporting of Indigenous status and cancer outcomes from cancer registries. The data attest to vast differences between regions across the world; furthermore, being Indigenous in a wealthy country such as Australia or Canada does not necessarily equate with better reporting or measurement of cancer outcomes. From a survey of 83 population-based cancer registries, Diaz et al^5^ report that only 66% collect Indigenous status information, with limited quality control for completeness and accuracy. Key barriers to collection of high-quality data are discussed.

In another article, co-authored by a multidisciplinary team of Indigenous and non-Indigenous researchers, Diaz et al^6^ analyze the impact of chronic disease in Australia’s First Peoples, Aboriginals, and Torres Strait Islanders, which underpins their 10-year life expectancy gap compared with non-Indigenous Australians. The nexus between vascular disease and cancer has spawned the new subspecialty of cardio-oncology; nowhere is this more prescient than for the Indigenous population. The links are multifaceted: common etiologies and shared risk factors; increased incidence of multimorbidity; cardiac dysfunction precluding or limiting anticancer therapies; and direct and indirect cardiac complications of cancer treatment. Each of these contributes to the disadvantages cancer outcomes. Common themes emerge in the original research by Sheppard et al,^7^ who report on the detrimental impact of pre-existing diabetes on survival after a breast cancer diagnosis in First Nations women in Ontario, Canada.

## Impact of Indigenous Culture

Research by Garvey et al^8^ reviews the four predominant issues reported in the delivery of psychosocial aspects of cancer care to Indigenous communities: patients’ experiences of care; supportive care needs; quality of life and well-being; and psychological distress. The research, as well as the research by Bastian et al,^9^ highlights the need for culturally specific tools for Indigenous patients to allow appropriate measurement of reported outcomes and experiences, quality of life measures, and supportive care needs.

As with clinical care, each aspect of conducting research, from ethics to communication to equity of access and translation into standard practice, is affected by beliefs and norms linked to ethnicity and culture. Ferrari^10^ presents bioethical and legal aspects of cancer contextualized by the Rondônia Indigenous populations of Brazil. This work emphasizes the importance of understanding the history of the peoples, including the impact of foreign settlement; the nexus between health, land, and territory; the need to appreciate differences between tribes and heritage groups, who may otherwise appear similar to an external party; and the role of traditional medicines.

The experience of Indigenous peoples with biobanking, tissue banking, and genomic research is presented in a literature review by Aramoana et al^11^ that summarizes 17 studies conducted across Alaska, Australia, Canada, New Zealand, Hawai‘i, and the United States. Four interdependent themes emerge: land, ancestors, culture, and bodily substances. These are postulated to arise through the strong cultural connection to ancestors and traditional lands. Biologic specimens are viewed as inseparable from these themes. The practical implications for researchers described by the authors render this compulsory reading for all cancer clinicians and researchers.

## Indigenous Empowerment

Letendre et al^12^ describe the evolution of the Canadian Indigenous Research Network Against Cancer, formed specifically to address disparities through advocacy and research priority setting by both Indigenous elders/knowledge holders and community voices. This was modeled on the Australian National Indigenous Cancer Network, of which Dr. Gail Garvey was a founding member. Similarly, Guerrero et al^13^ describe the establishment of The Partnership, developed to enhance cancer research capacity building through training and outreach at collaborating institutions for and by the Pacific Peoples of Hawai‘i, Guam, and the US Associated Pacific Islands.

Complementing the article on attitudes about biobanking, Caron et al^14^ describe the formation of the Northern First Nations Biobank Advisory Committee of the Northern Biobank Initiative in British Columbia, Canada. This was formed in response to Indigenous communities being “at best minimally represented and at worst actively excluded”^14(p1)^ from human tissue biobanks. Inclusion of diverse populations in cancer-biospecimen research not only enhances our ability to understand this disease more comprehensively but will likely allow development of treatment strategies for subpopulations that would otherwise be missed if their tissue was not included.

In an erudite commentary piece laying open the issue of the role of non-Indigenous researchers in performing research on Indigenous populations, Scott et al^15^ provide reflections and observations on the roles and responsibilities of non-Indigenous cancer researchers and identify some of the principles that guide researchers in Aotearoa, New Zealand. Noting that “most health research is relevant for Indigenous peoples, and most researchers are non-Indigenous,”^15^ the authors caution that, “if researchers don’t engage with Indigenous research principles, health research can and does result in significant harm for Indigenous peoples.”^15(p1)^

An impressive example of research underpinned by the principles outlined by this caution is the article by Ristevski et al,^16^ who present a deep analysis of the importance of Aboriginal peoples’ cultural and family connections to inform the development of culturally safe cancer survivorship models of care. Data were collected through yarning, a culturally appropriate methodology relevant to Indigenous Australians when sharing information, knowledge, and culture. One yarning circle used a talking stick that “when held, allowed that person to tell their story without interruption or comment.”^16(p2)^ In this way, experiences of Indigenous cancer survivors were articulated, including the impact of cancer diagnosis and treatment on self, family, and community.

Translation of the understanding of Indigenous culture into practical tools to enhance cancer outcomes is described by Chynoweth et al.^17^ The first population-specific optimal care pathway describing the principles and steps of optimal cancer care for Aboriginal and Torres Strait Islander People was developed during a 2-year, government-initiated engagement process involving more than 70 organizations and groups. The resource is designed for use by patients and their treating teams, to equip health services and clinicians to identify gaps in cancer services as well as to inform quality improvement initiatives.

It is not hard to guess that cancer in Indigenous peoples is a subject that we hold dear. As passionate advocates of global oncology. A movement to ensure equity of access for all people to cutting-edge cancer care and research, it is easy to see that Indigenous populations constitute important and diverse minority groups who suffer worse cancer outcomes. We must take responsibility for all peoples, because cancer is a global disease, to ensure cancer care is delivered equitably and respectfully around the world. In this era of precision or personalized oncology, understanding the patient must be considered as important as understanding the tumor. The topic of cancer in Indigenous populations provokes fear and confusion, arising from ignorance because of the paucity of information in this space. We hope this issue of *JGO* will raise awareness and ignite interest in this field, not just for those members of Indigenous populations but for all who seek to conquer cancer.
